# The adaptive significance of human scleral brightness: an experimental study

**DOI:** 10.1038/s41598-022-24403-2

**Published:** 2022-11-24

**Authors:** Slawomir Wacewicz, Juan Olvido Perea-García, Zdzisław Lewandowski, Dariusz P. Danel

**Affiliations:** 1 Center for Language Evolution Studies, Nicolaus Copernicus University, Toruń, Poland; 2 The Cognitive Psychology Unit, Faculty of Social Sciences, Leiden University, Leiden, The Netherlands; 3 Department of Human Biology, Wroclaw University of Health and Sport Sciences, Wroclaw, Poland; 4 Department of Anthropology, Hirszfeld Institute of Immunology and Experimental Therapy, Polish Academy of Sciences, Wroclaw, Poland

**Keywords:** Biological anthropology, Human behaviour

## Abstract

Homogeneously depigmented sclerae have long been proposed to be uniquely human—an adaptation to enable cooperative behaviour by facilitating interpersonal coordination through gaze following. However, recent evidence has shown that deeply pigmented sclerae also afford gaze following if surrounding a bright iris. Furthermore, while current scleral depigmentation is clearly adaptive in modern humans, it is less clear how the evolutionarily intermediate stages of scleral pigmentation may have been adaptive. In sum, it is unclear why scleral depigmentation became the norm in humans, while not so in sister species like chimpanzees, or why some extant species display intermediate degrees of pigmentation (as our ancestors presumably did at some point). We created realistic facial images of 20 individually distinct hominins with diverse facial morphologies, each face in the (i) humanlike bright sclera and (ii) generalised apelike dark sclera version. Participants in two online studies rated the bright-sclera hominins as younger, healthier, more attractive and trustworthy, but less aggressive than the dark-sclera hominins. Our results support the idea that the appearance of more depigmented sclerae promoted perceived traits that fostered trust, increasing fitness for those individuals and resulting in depigmentation as a fixed trait in extant humans.

## Introduction

Human eyes are exceptional: in addition to their unusual horizontal elongation, they have homogeneously pale sclerae, which makes them highly conspicuous and transforms them from sensory organs into signalling devices. However, the understanding of the exact role of this morphology has remained highly speculative, and it has not yet been systematically and comprehensively studied with a view to evaluating the variety of its possible underlying adaptive functions. Interest in this topic was first sparked with the early comparative studies on primate external eye morphology by Kobayashi and Kohshima^1,2^, which singled humans out as having a uniquely conspicuous coloration, and proposed that this coloration evolved to facilitate gaze-following and triadic communication^3,4^. Several influential accounts of human socio-cognitive evolution^3,5,6^ have followed this idea, with claims that dating the emergence of this conspicuous morphology “would suggest a possible date for the origins of uniquely human forms of cooperation and communication” (Tomasello et al. 2007: 319)^3^.

However, more recent research points to a broad variety of social and ecological factors that influence the diversity in external eye appearance in primates; for example, such diversity may be driven by the behavioral ecology of specific lineages, such as the spectral quality and quantity of light from the sun in the species’ range^7^. Furthermore, functional studies^8^ as well as simulations^9,10^ show that the eye-gaze of other primate species previously considered to have cryptic eye gaze is actually conspicuous, supporting the proposal by Perea-García et al.^11^ based on comparative morphological evidence. This is especially interesting in the case of chimpanzees, which are characterized by deeply pigmented conjunctiva, but whose typically bright amber irises create a stark contrast between these two tissues.

In sum, pigmented sclerae may also afford gaze following by onlookers, and we assume that scleral pigmentation is the ancestral state in primates—since most studied primate species do display some degree of scleral pigmentation^7^. This begs the question: if pigmented sclerae afford gaze following, why did depigmented sclerae become fixed in our lineage? The transition from pigmented to depigmented sclerae in humans may have been mediated by several mutually non-exclusive proximate functions^12^, among which the literature suggests the signaling of attractiveness and health (e.g. through symmetrical depigmentation that affords the perception of changes in vasculature or pigmentation), signalling of reliability and trustworthiness (e.g. by facilitating the perception of one’s gaze and emotions and, thus, intentions), and signalling of reduced emotional reactivity and reactive aggression (possibly sharing proximal mechanisms with self-domestication). In addition to affording signaling functions, the loss of populations of epithelial stem cells in the nasal and temporal limbus^13^ may have been a prerequisite for the extensive and homogeneous loss of pigmentation that characterizes our species. This is because the superior and inferior limbus are typically covered by the eyelids, protecting these cell populations from increased risk of UV damage that are essential to keeping a transparent cornea^14^ and, therefore, vision. By contrast, the presence of lateral stem cell populations may necessitate increased pigmentation to reduce the chances of keratopathy (or corneal abnormality)^15^.

We believe that a detailed account of the natural history of our extant eye appearance necessitates a holistic approach combining sources of evolutionary, environmental, developmental, behavioral and psychological evidence. In this study, however, we restrain our focus to the factors pertaining to the psychology of the differential perception of psychological traits on the basis of changes in scleral brightness. To start evaluating the relative contribution of these factors, we developed a novel rating design that allowed us to collect evaluations of humanlike faces with a bright versus dark sclera while keeping the participants unaware of the experimental modification. To this end, we morphed and photo-edited facial images of a range of reconstructed species of ancient hominins (see “Stimulus” below), to create realistic facial images of 20 individually distinct hominins with diverse facial morphologies; each face in the (i) “humanlike” bright sclera and (ii) “generalised apelike” dark sclera version. This stimulus was presented online to 250 (Study 1) and 100 (Study 2: self-replication) demographically diverse participants, who rated the individuals in the images on their perceived age, health, attractiveness, trustworthiness and aggressiveness.

### Predictions

We predicted that the bright-sclera hominin faces would be rated as younger, healthier, more attractive and trustworthy, and less aggressive than the dark-sclera faces.

#### Health

The external appearance of the sclera is known to change with a wide range of health conditions, making it a useful tool in medical diagnosis, and, by extension, a reliable cue to several aspects of an individual’s health status. The depigmented sclera in humans provides a blank canvas that showcases changes in vasodilation and blood flow through vessels of the conjunctiva^16,17^. The resulting differences in eye redness may indicate a range of underlying health issues, including inflammation, irritation and trauma of ocular structures^16,18,19^, subconjunctival haemorrhage^20^, glaucoma^19^, and more general and systemic conditions such as hypertension, diabetes^21^, sickle cell and autoimmune diseases^22,23^ as well as lack of sleep^24^ and intoxication^25–27^. A yellowish coloration of the sclera is a typical symptom of jaundice and high blood level of bilirubin, and may indicate multiple underlying diseases causing impaired bilirubin metabolism, liver dysfunction or biliary-tract obstruction^28^.

Several experimental studies showed that digitally manipulated redness and yellowness of the sclera affected the health perception of observed eye photographs. Compared to unmanipulated stimuli, individuals with redder and yellower eyes were perceived as less healthy^29,30^. Similarly, Caucasian female facial pictures with decreased eye redness and yellowness were perceived as healthier than identical portraits with eye colours manipulated in the opposite direction^31^.

#### Age

The range of cues that scleral coloration provides to an individual’s health partly overlaps with those indicative of the individual’s age. For instance, yellowing of the sclera, caused by fat deposition between collagen fibres over the years, is a visible sign of ageing^32,33^. In addition, age-related accumulation of lipids is visible in other eye structures, such as arcus senilis^34^, which can further enhance the impression of the eye having a yellowish coloration. Continuous exposure to sunlight and ultraviolet radiation, which causes colour changes in the elastic fibres in the conjunctiva, may be another factor causing the apparent yellowing of the sclera^35^. Similarly, it has been suggested that cumulative lifetime experience of health conditions affecting blood vessel dilation in the conjunctiva may result in a higher baseline redness in the eyes of older individuals^31^.

All the above factors may cause the eyes to turn less white with age^31,35,36^. Experimental studies show that people indeed correctly associate changes in eye colouration with increased age. Experimentally increasing the redness and yellowness of the sclera in digital photographs leads to the modified stimuli being perceived as older^30,31^, and individuals with brighter sclerae are perceived as younger than those with darker sclerae^30,31,35^.

#### Attractiveness

From the perspective of human mate choice, both good health and youthfulness indicate a higher reproductive fitness of an individual^37–39^. Therefore, both those attributes are important determinants of human mate value and are preferred in sexual partners^40^. As a result, sexual selection will favor visual markers of these qualities; that is, individuals displaying signals and cues of health and youthfulness—such as a white sclera—should be perceived as attractive and preferentially selected as sexual partners.

This prediction has indeed been confirmed in several experiments with digital manipulations of scleral coloration. For example, individuals with redder and yellower eyes were assessed as less attractive compared to control portraits with the sclera left unmodified^29–31^. By analogy, individuals with darkened sclerae were also perceived as less attractive than those with untinted eyes^31^. Importantly, scleral whiteness directly measured on individual and unmodified pictures of the eye region in women was also positively correlated with attractiveness ratings^35^. However, the positive effect of sclera whiteness on attractiveness may be limited. This is because enhancing sclera whiteness above the original values (i.e. having "super-white" eyes) did not increase ratings of attractiveness of the photographed eye regions^30^. In the context of sexual preferences, it is also worth noting that the attractiveness rating may be further modified by the sexually dimorphic nature of the ocular morphology. As recent findings show, both sclera shape^12,41^ and colour^36^ are sexually dimorphic at least in Caucasians. However, the link between attractiveness and differences in ocular morphology in men and women should be inspected in more detail, as it may be mediated by age: brighter sclerae are associated with youth, and younger people are perceived as more attractive.

#### Aggressiveness

A preference for eye-like stimuli in newborns^42^ and its subsequent development in neurotypicals, and more broadly, for specifically human patterns of complex sociality, may have built on selection against reactive aggression in our lineage. Reduced emotional reactivity to eye contact may have enabled the other signalling functions of the eye, and in particular the co-option of eye-gazing signals for purposes other than negotiating dominance in agonistic displays. The idea that scleral depigmentation could be a by-product of selection against aggression was proposed by Perea-Garcia et al.^11^. The authors showed that humans and bonobos (*Pan paniscus*) have relatively bright sclerae compared to common chimpanzees (*Pan troglodytes*). Such observation, later confirmed by other authors^43^, led to the question of why closely related species evolved different patterns of eye colouration. Selection against aggression in humans^6^ and bonobos^44^ is one candidate answer. The self-domestication hypothesis^44^ proposes that many of the traits that characterize humans (increased fecundity from an earlier age, reduced reactive aggression, increased period of socialization, extended weaning periods, reduced facial prognatism) are by-products of selection against aggression, similarly to a suite of seemingly unrelated traits that appear in domesticates compared to their wild counterparts^45^. When compared to the wild-type, depigmentation is one of the most commonly found features in domesticated animals^45,46^^.^ This is due to the shared embryogenic origin of melanoblasts and the adrenal medulla^45^. Thus, with selection for reduced emotional reactivity, reduced pigmentation is also indirectly selected. While scleral depigmentation may be originally a functionless byproduct of selection against aggression, it is possible that bright sclerae became a marker of reduced emotional reactivity, which could then itself become target of selection, as it would reliably announce less emotionally reactive individuals^11^. More direct evidence for the link between scleral correlation and actual intraspecific aggression comes from a recent study by Mearing et al.^47^, which reports a significant negative association between scleral brightness and conspecific lethal aggression in 108 primate species.

#### Trustworthiness

Finally, a body of evidence supports the idea that the whiteness of the human sclera functions to advertise gaze direction as well as a range of information regarding the internal state of the owner of the eyes. As is best illustrated by the wide utility of eye-tracking technology in behavioral research, gaze is a rich, reliable and relatively easily observable source of information on an individual’s cognitive processing, including mindfulness and cognitive load^48^^,^^49^, but in particular one’s attentional states. Naturally, all this valuable information can be accessed by conspecifics and other animals, and potentially exploited in competitive contexts. Indeed, apes have been shown not only to make use of gaze cues in competitive foraging^50^^,^^51^, but also to be aware of the information carried by their own gaze, and occasionally to use own gaze direction strategically to mislead competitors^52^. Similarly, humans are aware of the social information that is leaked through their gaze direction, and make strategic decisions on what to look at, depending on whether or not they believe their gaze is visible to third parties^53–56^. In short, gaze is one of the basic predictors of immediate behavioural intent that humans use in interaction, including emotionally based intent (such as approach vs avoid, cf. Adams and Kleck^57^) and aggressive action (such as in hand-to-hand combat, cf. e.g. Hausegger et al.^58^).

The depigmented sclera characteristic of humans functions to advertise gaze direction, helping conspecifics infer one’s attentional states, which has been proposed to facilitate cooperation. Kobayashi and Kohshima (2001: 433)^2^ proposed that “gaze-signal enhancement might aid the conspecific communication required for increased co-operative and mutualistic behaviours to allow group hunting and scavenging”, an insight later developed into the “cooperative eye” hypothesis^3^^,^^59^, which claims that enhanced gaze-following would have been adaptive by facilitating coordination and thus increasing the effectiveness of collaborative subsistence tasks^4^. There is however a complementary account of the relation between a depigmented sclera and cooperativeness, not focusing on the efficiency of cooperative action but instead on evaluating the reliability of the cooperative partner. As is well-known, cooperation is typically not an evolutionarily stable strategy^60^, because despite the benefits it might bring in the long term, it is typically outcompeted by defection in the short term. Partner choice is therefore key, since cooperation is only profitable with reliable partners, i.e. such that one can trust to act as cooperators rather than defectors^61^. A depigmented sclera makes one’s gaze patterns easier to observe, thus facilitating inferences about that individual’s knowledge, attention and intentions that are crucial in predicting behavior. By making the individual more readable and easier to monitor, a depigmented sclera could serve as an advertisement of their honesty, trustworthiness, and reliability. Humans indeed routinely use gaze information in assessing the trustworthiness of interactants, and in particular strongly associate gaze aversion with dishonesty. Despite a lack of evidence in its support, the belief that “liars avert gaze” is in fact the single most dominant “pan-cultural stereotype” about liars^62,63^. Interestingly, a recent bottom-up study into facial correlates of perceived trustworthiness^64^ identified a darkened pupil-iris area (thus, increased perceptual contrast with the surrounding white sclera) as the only facial feature stably cross-culturally correlated with higher trustworthiness judgments.

## Study 1

We developed a rating study with the faces of artificially generated ‘generalised hominins’, which allowed us to collect evaluations of humanlike faces with a bright versus dark sclera while keeping the participants unaware of the experimental modification. The hominin facial morphologies used in the study made the embedding of both sclera variants (i.e. bright, humanlike vs dark, apelike) look sufficiently natural that it did not attract conscious attention from the participants. Equally, the facial morphologies were sufficiently humanlike for the participants to feel natural rating this stimulus on human attributes. Our study was conducted in accordance with the principles of the Declaration of Helsinki. Ethical approval for this study was obtained from the Scientific Research Ethics Committee at the University of Warmia and Mazury in Olsztyn (decision 16/2021). Our study was pre-registered with the AsPredicted service at https://aspredicted.org/TM4_PSK

### Stimulus

**Figure 1:**
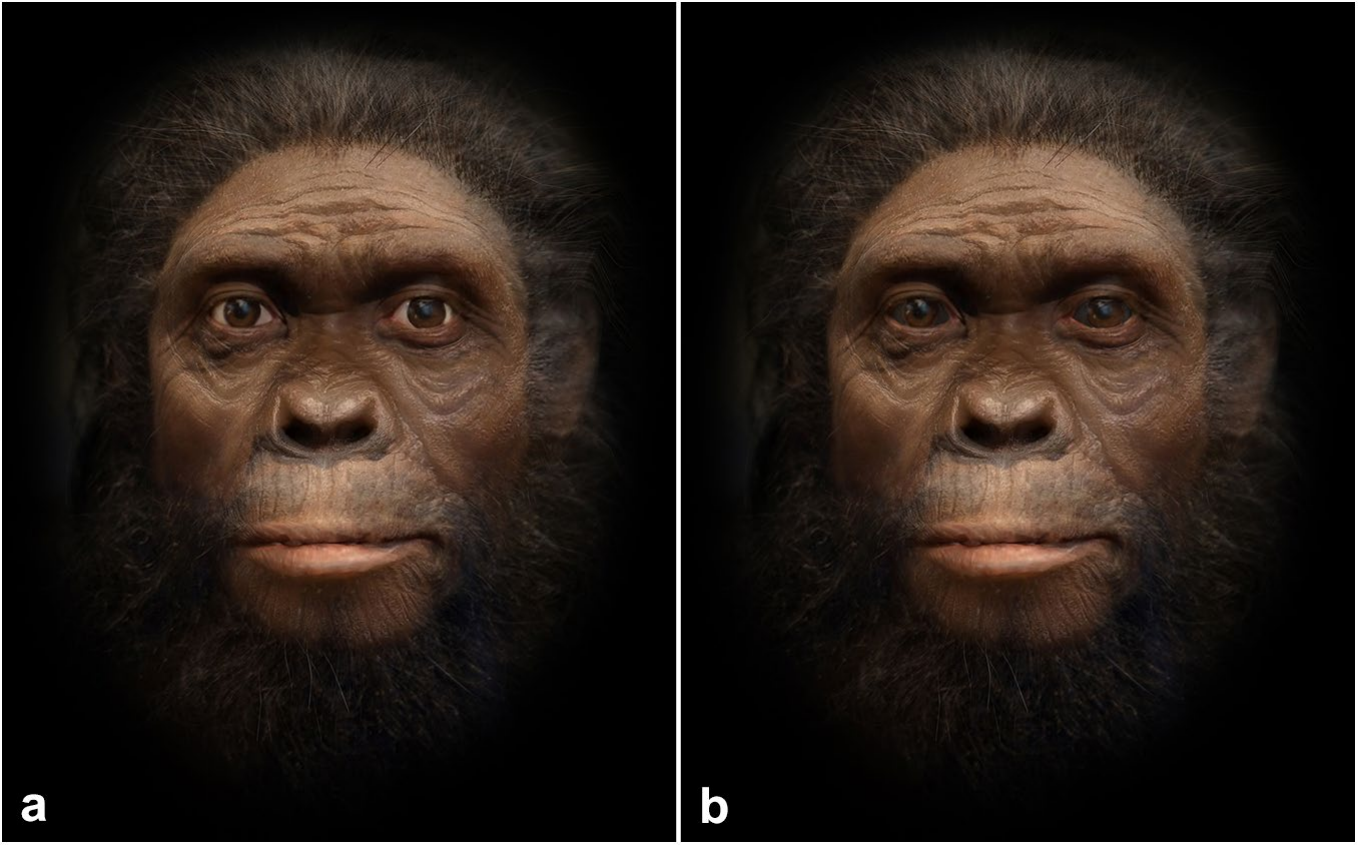
An example facial image used as stimulus in the bright sclera (left, + 50 PS brightness) vs dark sclera (right, −150 PS brightness) versions. Artwork by ZL, through digital editing of the following images: Fig. 7 by Campbell RM, Vinas G, Henneberg M and Diogo R (2021)^76^—https://www.frontiersin.org/articles/10.3389/fevo.2021.639048/full. *Homo habilis* by Cicero Moraes—https://commons.wikimedia.org/wiki/File:Homo_habilis_-_forensic_facial_reconstruction.png. *Homo heidelbergensis* by Sam_Wise—https://www.flickr.com/photos/sortingoutscience/5071085511/sizes/o/.

Stimulus in our study was a set of 20 artificially created realistic facial images of generalised ancient hominins, each in a bright sclera and dark sclera version. As source images we used 31 medium- to high-quality facial images of reconstructions of a range of extinct hominin species (see Additional online material for more details about those images, https://osf.io/7cr9e/?view_only=49f985066f77441fb29e015aa077c6a3), acquired through targeted internet searches. The "Facemixer" utility in the Abrosoft FantaMorph Deluxe for Windows software was applied to mix the features and shapes of the source faces; typically three to four input faces were used to compose an individual output face, which made it possible to retain considerable individual detail of the input faces. Each of the resulting output images was corrected by an artist to preserve the visual uniformity of the set. Photoshop CC was used to eliminate artefacts, introduce specific corrections such as closing a slight opening of the mouth, and add a black mask around the face; each image was edited individually. The Lightroom CC software was used to improve the overall visual quality of the images. This resulted in 38 realistic facial images of individually distinctive ‘hominins’ portrayed en face, looking straight and with a neutral facial expression. Of these, 20 were selected to form the final set to maximise individual differences between the images. Finally, the brightness of the scleral areas of each facial image was adjusted by +50 Photoshop brightness points to create a “humanlike” bright sclera and, depending on the image, by either −150 or −200 points to create a “generalised apelike” dark sclera version (Fig. 1). Extensive piloting that preceded the study confirmed that in an overwhelming majority of cases, the experimental manipulation is not detected by the participants.

### Procedure

250 demographically diverse participants (aged 18–61, mean 26.16; from 43 countries of birth, 28 countries of current residence—see Additional online material for more detailed demographic data of the participants and for the design implementation) informedly consented to taking part in the experiment through the online crowdsourcing platform Prolific. Eligibility criteria were set to having completed at least two previous Prolific studies, 100% approval rate on previous studies, and declaring normal or corrected to normal vision as well as fluency in English. Participants could use desktop or laptop computers, but not tablets or smartphones. Participation was remunerated (1 GBP). The study was implemented with the Labvanced online application. Each participant was shown 10 bright-sclera and 10 dark-sclera faces, presented in random order. The stimuli were described to the participants as “the faces of prehistoric ancestors of modern humans (so-called hominins, i.e., "apemen" or "cavemen")”. Each participant saw the faces of all the 20 hominins in the set, but only one condition per face; that is, participants saw each face only once, with either bright or dark sclerae. Participants rated the faces on five dimensions—age, health, aggressiveness, attractiveness (“to others like her/him”), and trustworthiness—by moving sliders on a horizontal line between the left (0) and right (100) extremes for each trait. No time limit was set for completing the ratings. The extremes of the scale had verbal labels (e.g. “very young” and “very old” for Age), but the underlying numerical values were not made visible to the participants. This assessment method drew on the adaptation of the visual analogue scale. It provided a large spectrum of possible responses, which allowed the detection of minute changes in the ratings while assessing multiple attributes and minimized the clustering of ratings around one value, as could be the case with categorical scales.

### Analysis

First, we excluded participants who did not complete the task, or whose completion time was shorter than Q1—1.5 × of the interquartile range (IQR) in our sample (the other planned exclusion criterion, “not moving the slider on any 4 or more consecutive ratings”, was not applied due to this being common in our sample). We excluded 31% of the original data set (n = 82), after which the rest of respondents (N = 168) were included in analyses. We fit separate generalized mixed models (GLMM) for each of our dependent variables (except *Age*, see below) with our manipulation (dark/bright sclerae) as an independent variable. For age, we fit a linear regression model (LM). We included stimuli pairs and participants as random effects in all our models. Through a software glitch, one of the faces in the bright sclera version was never displayed to participants (this was corrected in Study 2). We predicted that bright-sclera hominin faces would be rated as less aggressive, more attractive, more trustworthy, healthier, and younger than the same faces with a dark sclera.

### Study 1: results

All models found statistically significant differences between pairs of stimuli in the predicted direction. These models are summarized in Table 1 below.

**Table 1:** Summary of the models for each of our dependent variables.

	Intercept	Dark–bright difference	t/z value	p
Age	63.13	4.03	5.30	< 0.01
Aggression	−0.44	0.28	38.369	< 0.01
Attractiveness	−0.19	−0.37	−50.76	< 0.01
Health	0.51	−0.39	−53.17	< 0.01
Trustworthiness	0.24	−0.31	−43.25	< 0.01

The model for age is a linear regression with error structure. All other models are generalized linear models with error structure.

## Study 2

Study 2 was conducted as a self-replication study to address minor limitations detected in the design of Study 1.

### Study 2: design and procedure

The stimulus and procedure were the same as in Study 1, with the following revisions.

All the stimulus images were equalised for overall brightness level; that is, the overall image brightness was made constant within each bright sclera–dark sclera pair of stimuli.

Participants were asked to complete a screen calibration procedure to ensure a proper rendering of the color scale on the display devices. Small changes were introduced to the phrasing of the instructions for participants, to further ensure compliance with the instructions. The only exclusion criterion for responses was time-based: we excluded all trials completed below 5 s, i.e. where the total time for providing all five ratings for a particular facial image was shorter than 5 s (i.e. less than 1 second per rating). Finally, the Labvanced application settings were corrected so as to ensure that all stimuli items were displayed and that the demographic data of the participants were associated with their responses. We further aimed to increase the demographic diversity of the sample by recruiting four batches of 25 participants, at times 4:00, 10:00, 16:00, 22:50 CET, so as to make the study equally available to Prolific users across the different time zones (total 100 participants; aged 19–74, mean 28.85; from 20 countries of birth, 12 countries of current residence—see Additional online material for more detailed demographic data of the participants). This self-replication study was pre-registered with the AsPredicted service at https://aspredicted.org/blind.php?x=GCY_FXV.

### Analysis

We excluded participants that did not complete the task, and we excluded all trials completed in less than 5 s (approximately 1% of all trials). Lastly, we solicited general feedback on the task through two post-study questions: "Did you notice anything particular about any area in the faces that influenced your decisions?", and "Do you have any other comments or other helpful information?". We conducted separate analyses for groups of participants categorised by their mentions of the eyes in the feedback comments: (a) eyes not mentioned at all or in an irrelevant way (e.g. “eyebrows”, “scar under the eye”); (b) eyes mentioned in general (e.g. “I think the eyes influenced my response”), or; (c) specific mention of our IV (e.g. “the darkness of the eye/sclera influenced my response”). These additional results are included in the Additional online material (https://osf.io/7cr9e/?view_only=49f985066f77441fb29e015aa077c6a3)—the results are qualitatively comparable to the main results we present here. We fit separate generalized linear mixed effects models (GLMM, g*lmer* function in the package *lme4* of R) for each of our dependent variables (except *Age*, see below) with our manipulation (dark/bright sclerae) as an independent variable. We included participants as random effects in all our models to allow the intercept to vary for each of them. For *Age*, we first fit a simple linear regression model (*lm* in base R) to inspect the residuals. Upon confirmation that the residuals conformed to assumptions, we fit a linear mixed effects model (LMM, *lmer* function in *lme4*, R) with *Age* as dependent variable and our manipulation as the independent variable.

### Study 2: results

**Figure 2:**
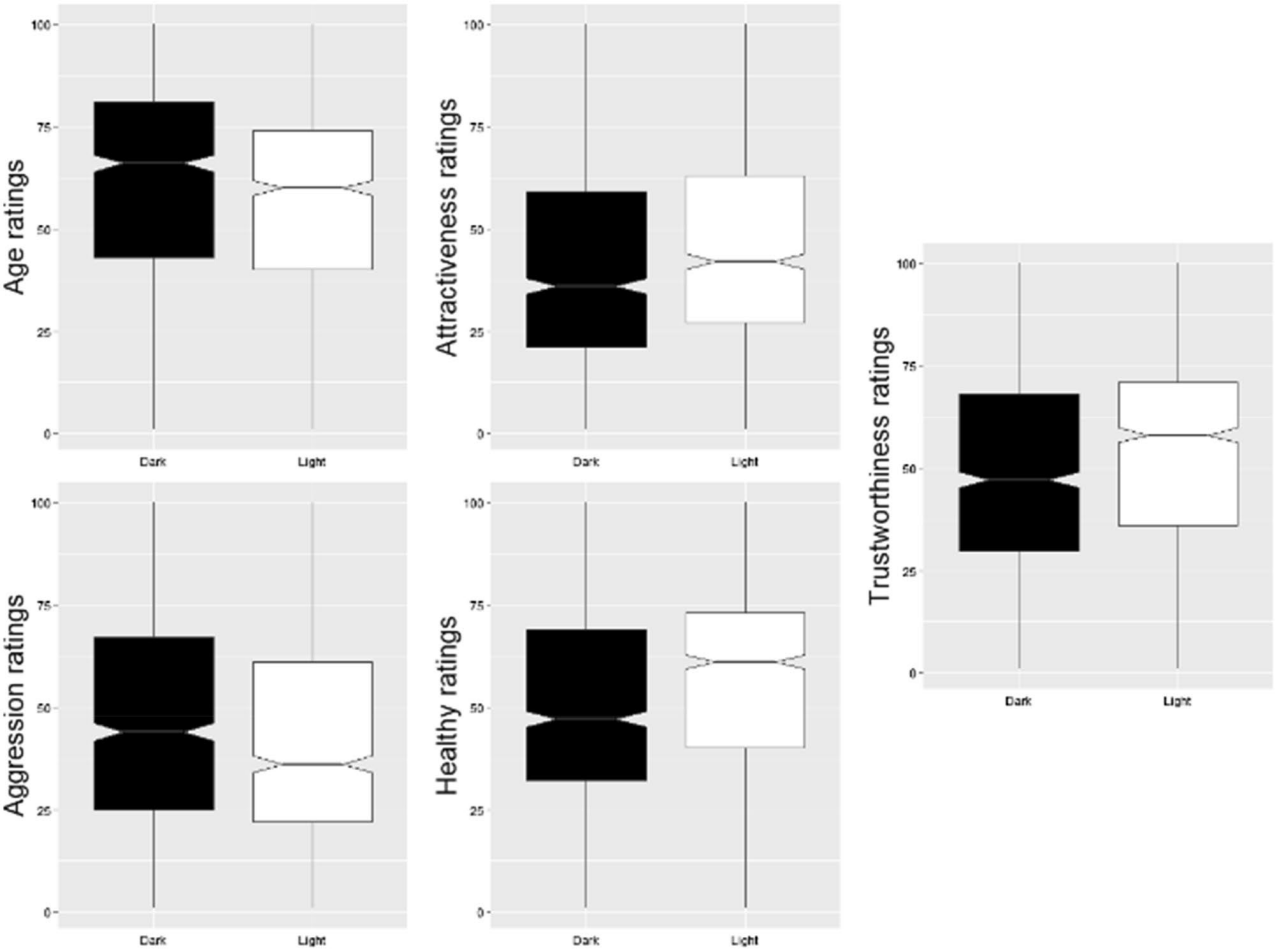
Comparison of ratings for each dependent variable in stimuli with dark or bright sclera. Whiskers represent range. Middle lines represent the median. Notches show confidence intervals (median + /− 1.58*IQR/sqrt(n)).

All models found statistically significant differences between pairs of stimuli in the predicted direction. Boxplots with a comparison in ratings between pairs of stimuli for each variable are shown in Fig. 2. These models are summarized in Table 2 below.

**Table 2:** Summary of the models for each of our dependent variables.

	Intercept	Dark–bright difference	z/t value	p
Age	62.41	4.88	4.78	< 0.01
Aggression	−0.12	0.26	26.89	< 0.01
Attractiveness	−0.49	−0.23	−22.96	< 0.01
Health	−0.01	−0.33	−33.92	< 0.01
Trustworthiness	−0.07	−0.23	−23.33	< 0.01

The model for age is a linear regression with error structure. All other models are generalized linear models with error structure.

## Discussion

The results of our experiments provide the first direct experimental evidence that a heavily depigmented scleral appearance typical of *H. sapiens* is associated with the perception of youth, health, attractiveness, trustworthiness and reduced aggressiveness relative to a pigmented scleral phenotype more typical of other ape species. This extends existing findings that subtle changes in scleral coloration are interpreted as cues to several parameters such as age, health or emotional state^30,31,35^, or that human infants have a generalised preference for a humanlike, bright scleral morphology^42^, and five-year-olds prefer the human morphology when choosing cooperative partners^59^. The effects we observed are robust: in particular, the self-replication and post-study feedback eliminate alternative interpretations related to factors such as participant pool composition, overall stimulus brightness, lack of direct control over participants’ display settings, and conscious biases related to the IV.

Our results lay the grounds for an interpretation that the transition from pigmented to depigmented sclerae in the ancestors of *H. sapiens* could be mediated by several convergent selection pressures related to the signalling of multiple parameters. Of course, drawing evolutionary conclusions from the results of laboratory studies with modern humans implies a degree of acceptance of the uniformitarian assumption, whereby the preferences (and with them, selection pressures) detected now can be extrapolated into the past. However, from an evolutionary perspective, it is coherent to think that certain psychological mechanisms selected in the past to aid in the survival and reproductive success of early hominins may have been inherited by modern humans, and now play a role in the perception of social-environmental stimuli. Such uniformitarian assumptions are foundational to a large body of human-evolutionary research^65,66^.

A more general limitation of our study is with interpreting our results as being directly caused by the specific constructs rated. One point relates to the correlations between the variables measured; for example, higher ratings of trustworthiness and non-aggression might reflect a spill-over or halo effect from attractiveness^67,68^. Some of our ratings are indeed highly correlated (see Additional online material). This could be due to the interrelatedness between the dimensions in real life, or they may all reflect a unitary positive construct. This can be illustrated with Perea-García et al.’s^11^ finding that sclerae become more highly pigmented throughout development in both chimpanzees and bonobos, suggesting that human scleral depigmentation is a neotenic or paedomorphic trait. As such, scleral depigmentation could signal youth, which would be related with health and attractiveness. Another point is the potential intermediary effect of stimulus familiarity. On the one hand, the fact that there are no a priori reasons for familiarity to influence (at least) the ratings of age and health makes us believe that this effect may be limited; on the other, this should be investigated by further studies, for example by incorporating an additional measure of familiarity.

An important limitation in making evolutionary interpretations lies in establishing the chronology of adaptive functions, i.e. distinguishing between the original adaptive function of the complete or partial depigmentation of the sclera and the functions that later co-opt this original structure. For example, the signalling of reduced aggressiveness and greater trustworthiness, and the signalling of age, health, attractiveness may have been shaped, respectively, by selection pressures for cooperative partner choice and sexual selection, which were proposed to be prominent at different points in the evolutionary history of our species^44^. Likewise, the involvement of irido-scleral contrast in triadic communication^4^ may not have been the most basal driver of selection for scleral depigmentation as proposed by Tomasello et al.^3^, but the result of co-option. As we have no way of reaching into the past to interview our extinct ancestors, answers to such questions can only be approximated through comparative research with other animals, in particular other primates^11,14,43,47,69–71^. The recent spike in such studies highlights the importance of comparative research in assessing the adaptive significance of human ocular morphology. While far from conclusively supporting that our ancestors perceived differences in scleral brightness the way our participants did, our results support said notion.

Lastly, there is a notable disconnection between studies on low-level visual perception and investigations on the perceived properties of individuals with more or less pigmented sclera. This disconnect may in part be due to the notion that, in humans, eyes constitute a special stimulus that is processed in unique ways^72^. It may be fruitful in terms of explanation to link both fields. For example, the perception of the contrast between the iris and sclera may be understood in terms of the Helmholtz illusion^73^ or, more specifically, as a result of centre-surround antagonism^74^.

In conclusion, we see our study as a methodological contribution to this fast-developing field. Interest in the adaptive significance of human ocular morphology was sparked a quarter-century ago with the influential study by Kobayashi and Kohshima^1^, and has now been revived with several very recent publications^12^. This particular wealth of studies within the last few years shows not only that this is a very timely topic, but most importantly testifies to the urgency of discussion on developing methodological standards. Our approach of using artificial humanlike facial images that represent no extant human population complements existing approaches that rely on humanlike stimuli which however fall outside of naturally occurring phenotypes^59,75^. This could be especially beneficial for future research programmes on the analyses of cross-cultural and socio-demographic effects on the perception of variability in ocular morphology.

### Ethical approval

Ethical approval for this study was obtained from the Scientific Research Ethics Committee at the University of Warmia and Mazury in Olsztyn (decision 16/2021).

## Data Availability

The datasets generated and analysed during the current study are available in the OSF repository, https://osf.io/7cr9e/?view_only=49f985066f77441fb29e015aa077c6a3.
